# Premise Plumbing “CT” (PPCT) as a Control Parameter for Opportunistic Pathogens

**DOI:** 10.1021/acsestwater.6c00582

**Published:** 2026-07-23

**Authors:** Tolulope O. Odimayomi, Darel C. Snead, William J. Rhoads, Myra D. Williams, Joseph O. Falkinham, Amy Pruden, Marc A. Edwards

**Affiliations:** † Via Department of Civil and Environmental Engineering, 1757 Virginia Tech, Blacksburg, Virginia 24061, United States; ‡ Department of Biological Sciences, 1757 Virginia Tech, Blacksburg, Virginia 24061, United States

**Keywords:** CT inactivation, *Legionella pneumophila*, *Mycobacterium avium*, premise plumbing

## Abstract

The product of disinfectant concentration and time (CT) is widely applied as a key pathogen inactivation parameter during potable water treatment. Here, we introduce the concept of premise plumbing CT (PPCT) as a part of an integrated framework for the control of opportunistic pathogens (OPs), such as *Legionella pneumophila* and *Mycobacterium avium*. A method is proposed for calculating CT at a given location in premise plumbing during stagnation events and demonstrated at scale in a chloraminated premise plumbing rig. Chloramine decay rates in stagnant PEX pipes were 2–3 orders of magnitude greater than in glass jars and dependent on initial chloramine concentration, contact time, temperature, pipe diameter, and flow rate. PPCT varied from 0 to 408 mg min/L from one pipe segment to another. No culturable *L. pneumophila* was detected in the test system pipes with PPCT > 78 mg min/L, which is comparable to reported CT thresholds for biofilm-associated *L. pneumophila* inactivation. Extending the concept to a lab-operated water heater yielded an estimated PPCT of 342.9 mg min/L, but robust growth of *M. avium* occurred in the tank up to 4 log CFU/mL, indicating extreme chloramine resistance. The PPCT framework introduced herein could provide a fundamental benchmark to help control OPs in the premise plumbing.

## Graphical abstract

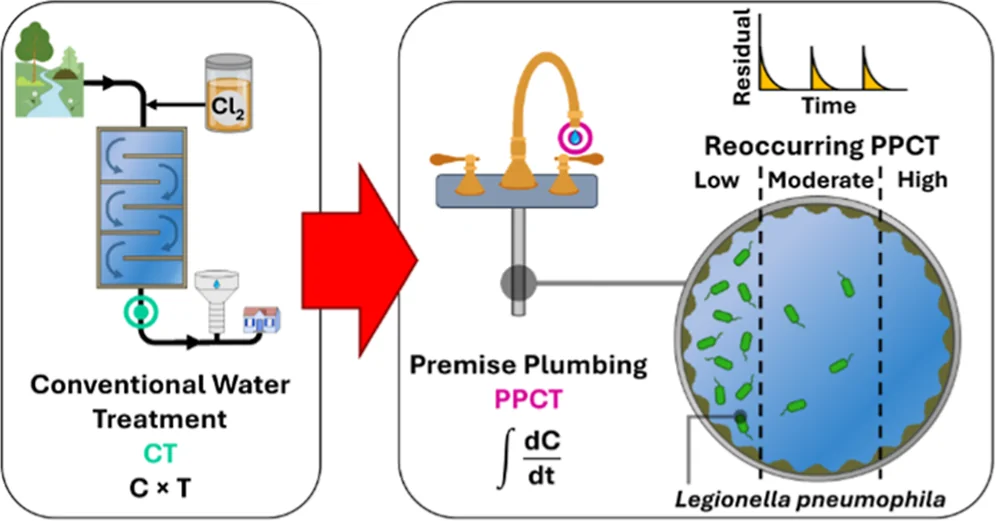

## Introduction

The Chick-Watson model for predicting bacterial and viral inactivation by disinfectants has been successfully applied for over 100 years.^1,2^ The product of a disinfectant concentration “C” and reaction time “T” (commonly known as “CT”) is used to estimate log removals of pathogens in the U.S. Environmental Protection Agency Surface Water Treatment Rule.^3^ For instance, the CT required to achieve at least a 3-log inactivation of Giardia cysts and a 4-log inactivation of viruses as a function of temperature, pH, disinfectant type, and disinfectant concentration was illustrated for a treatment plant by Clark and Regli, 1993.^4^

Public water utilities use CT to determine removal credits as water flows through the treatment plant and distribution system prior to the nearest customer.^5^ CT values for regulatory compliance were derived from bench-scale experiments, often employing chlorine-demand-free buffer solution in nonreactive containers or using pure cultures of pathogens exogenously spiked into test containers under conditions that are relatively inhospitable to their survival.^3,6–9^ That data is adjusted to correct for nonideal disinfection kinetics and flow patterns when applied to real water treatment plants and distribution systems.^5^

Published CT values would also need to be altered to account for the premise plumbing environment, where mixed microbial community biofilms are prevalent and pathogens that are disinfectant tolerant or resistant can grow.^10–17^

Opportunistic pathogens (OPs), including *Legionella pneumophila* (*Lp*) and *Mycobacterium avium* (*Ma*), are now the leading source of drinking water-associated disease and death in the US and many parts of the world,^18,19^ and their inactivation in premise plumbing by residual disinfectants is not yet predictable.^11,20–27^ Here, we introduce the premise plumbing CT (PPCT) concept, adapting the traditional CT concept to premise (i.e., building) plumbing, where it could prove invaluable toward improving guidance for the control and management of OPs.

Conceptually, any secondary disinfectant residual that crosses the property line and persists in the premise plumbing could help control pathogen growth in premise plumbing systems. If a sufficient concentration of disinfectant is maintained for a sufficient time (i.e., CT) daily or weekly at a given point of a premise plumbing system, pathogens might be controlled, even if other circumstances favor growth. However, a myriad of factors encountered in premise plumbing can accelerate the decay of disinfectants, potentially making a one-time residual measurement at the tap insufficient for characterizing microbial inactivation capability. Such factors include stagnation, reactive pipe materials, microbial activity, temperature, and sediment. In addition to enhancing microbial growth and providing a habitat for OPs,^28,29^ sediment can accelerate disinfectant decay.^30^ If the net effect of such factors could be accounted for, adjustments could be made to the PPCT framework to improve the prediction of inactivation. Such adjustments are analogous to that applied to fecal pathogen inactivation at the treatment plant, where CT corrections are commonly applied for pH, colder temperatures, and the presence of protective particles.^31^

This study introduces and tests a methodology for the newly proposed PPCT framework. PPCT is calculated in pipe segments representing a range of scenarios in an at-scale premise plumbing pipe rig operated over several months at variable temperature and 0–2.5 mg/L chloramine influent as Cl_2_. The relationship between estimated PPCT and measured levels of *Lp* and *Ma* is then examined in the context of situations leading to nondetectable, moderate, or elevated levels of growth. These PPCT values are also compared to CT values reported for these microorganisms under idealized conditions.

## Methods

### At-Scale Plumbing Rig Configuration and Operation

Premise plumbing was simulated with an at-scale rig with eight cold water and eight hot water 134-foot PEX-B pipe loops (SharkBite, Atlanta, GA) that were hard-plumbed to a chloraminated public water supply. Cold water flowed directly to cold pipes while hot pipes received water that had been warmed in a 72 L (19-gallon) electric water heater at a set point of 40 °C, which is within an ideal range for *Lp* growth in premise plumbing. Granular activated carbon (GAC) filters (Pentair, Golden Valley, MN) were installed upstream of the rig in a manner that permitted adjustment of the ratio of the municipal water and GAC-filtered water. Adjustment of this ratio provided a means to control the disinfectant residual in the water entering the rig, such that 0% GAC-treated water corresponded to the ∼2.5 mg/L Cl_2_ residual in the public water supply and 100% GAC-treated water provided a residual as low as <0.05 mg/L Cl_2_. Target influent chloramine concentration was sequentially increased during each phase of this study: <0.2, 0.25, 0.5, 1.0, or ∼2.5 mg/L (Figure 1). The room temperature was also controlled to compare 18 to 25 °C. The pipe rig contained mature biofilms aged 4–6 years at the time of the experiments described herein. At least one month of acclimation was allowed between each influent residual target adjustment before collecting representative chemical and biological samples. Under normal operating conditions, solenoid valves (ASCO Red Hat, St. Louis, MO) run by automated timers (ChronTrol, San Diego, CA) were used to flush each pipe once a day for 35 s. The cold water influent (i.e., blend of unfiltered and GAC-filtered city water) was purged for 10 min prior to the daily flushing to ensure that water entering the rig was at steady state and did not have unusually high levels of microbes.

**Figure 1:**
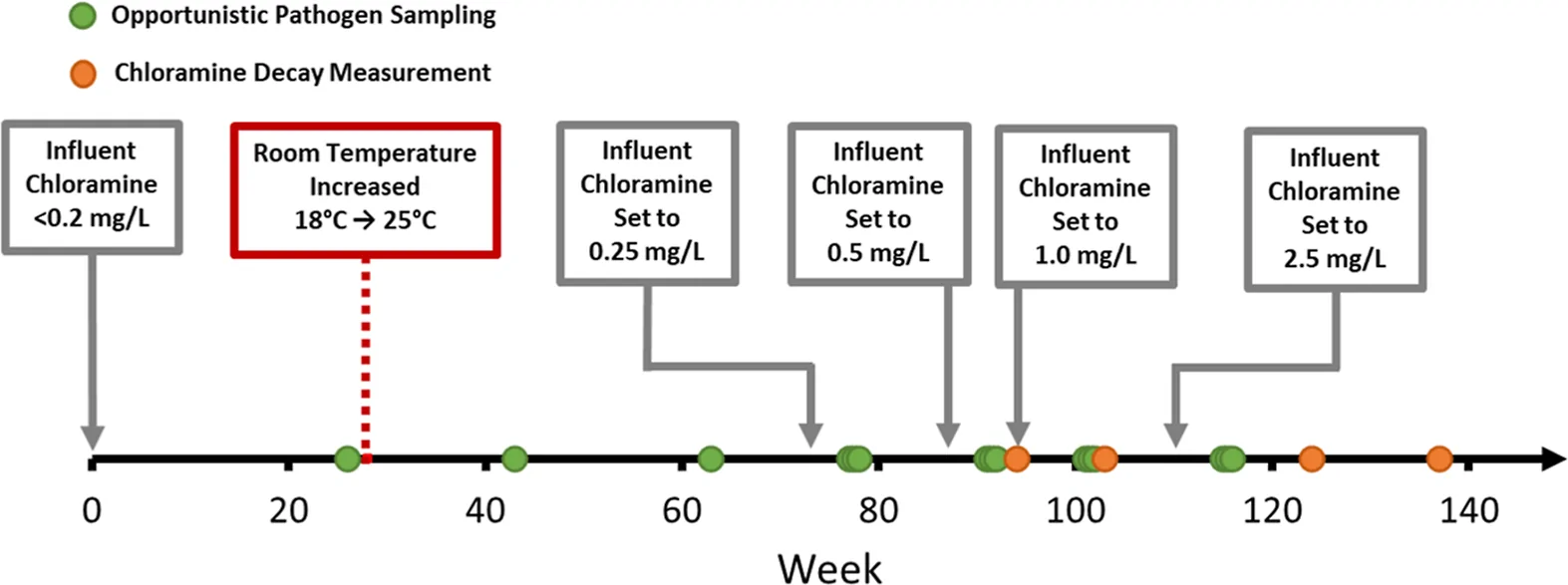
Experimental timeline of adjustments to influent chloramine and room temperature. Opportunistic pathogens *L. pneumophila* and *M. avium* were measured at all taps three times per influent chloramine condition. Chloramine decay profiles and “CT” were measured in cold 1 day WRT taps during the 0.5, 1.0, and ∼2.5 mg/L influent chloramine conditions.

The rig was equipped with 30 sampling locations: the cold water influent, influent water after it passed through the water heater (3.87 day hydraulic retention time), 14 different cold water pipe tap configurations, and 14 congruent taps distributing hot water. The 28 different cold and hot water taps had flow rates of either conventional faucet flow at 8.3 L/min (2.2 gpm), moderate flow at 5.7 L/min (1.5 gpm), or extremely low flow at 0.95 L/min (0.25 gpm). Pipe diameters ranged from 6.35 to 19.05 mm (0.25 to 0.75 in.), and the resulting flow velocities were 0.07 to 1.16 m/s (0.22 to 3.82 fps). These plumbing conditions resulted in water retention times (WRT) at the taps that ranged from 1 to 21.3 days, allowing exploration of extremes commonly encountered in real-world premise plumbing. A field blank was processed alongside all samples. Details on the rationale for the design and consumer water use scenarios replicated by the rig are presented in our previous study.^32^ Briefly, each pipe had a tap at the terminal point 40.84 m from the entry point of the pipe. All pipes also had a tap at a point along the length of the pipe where the WRT was 1 day, representing “right-sized” plumbing that adjusts length based on flow rate and pipe diameter. The rig acclimation, OP colonization, and water heater sediment testing are discussed in Supporting Information SI 1 and 2.

### Chloramine Measurement and Decay Profiling

Residual chloramine was measured at each sampling location before and after the daily flush cycle for influent chloramine levels of 0.5, 1.0, and ∼2.5 mg/L to evaluate which taps had a residual favorable for decay profiling. To determine decay profiles, each 1 day WRT cold water taps was first flushed with 2–3× the normal daily volume to achieve initial chloramine levels equal to the influent. As the water stagnated in the pipes, 15 mL aliquots (0.2–1.2% of the total pipe volume) were drawn from taps hourly to measure residual disinfectant.

### Premise Plumbing CT (PPCT)

PPCT values were approximated as the area underneath the decay curve of chloramine concentration at the tap versus stagnation time (Figure S1; eq S1). This approach differs from traditional CT calculations used for regulatory compliance at a treatment plant, which takes the minimum residual detected at the end of a treatment process and applies it as a constant value throughout the time of treatment. This assumption is not transferable to buildings where disinfectant can decay quickly or reach undetectable levels. Here, if chlorine fell to nondetectable levels (≤0.03 mg/L) within the first 8 h, PPCT was calculated up to 8 h. If a residual persisted above 0.1 mg/L Cl_2_ after 8 h, the remaining CT was calculated up to the time when the residual dropped below 0.1 mg/L (see detailed explanation in Figure S2; Table S1). The calculated PPCT values were then compared to measured *Lp* and *Ma* concentrations. Threshold PPCTs where OPs were effectively suppressed were compared to reported CT thresholds derived from standard bench-scale experiments. Details on modeling PPCT in the rig are provided in Supporting Information SI 3 and eqs S2–S5.

### Disinfectant Decay in Glass Jars

Disinfectant decay rates were also determined in 250 mL glass jars without biofilm or pipe at 5–39 °C as a control to elucidate the impact of the plumbing, associated biofilm, and temperature on decay rates observed in pipes. Details of the glass jar experiments are provided in Supporting Information SI 4.

### Chemical and Microbial Analysis

A HACH DR3900 spectrophotometer was used to measure total chlorine with DPD at a detection limit of 0.02 mg/L (method 8167, HACH, Loveland, CO). To acknowledge that the municipal water supply feeding the rig is chloraminated, total chlorine measurements are herein termed in text as chloramine. Some level of organochloramines with reduced disinfection efficiency may have been present at low levels in the supply water. Total cell counts were measured using flow cytometry (BD Accuri C6; BD Biosciences, San Jose, CA) after staining samples with SYBR Green I (Invitrogen, Carlsbad, CA) fluorescent nucleic acid. Culturable *Lp* was quantified using the 100 mL potable IDEXX Legiolert protocol (IDEXX, Westbrook, ME), and culturable *Ma* was enumerated through spread plating on Middlebrook M7H10 medium containing 0.5% (v/v) glycerol and 10% (v/v) oleic acid-albumin. The detection limit of the Legiolert samples was 0.01 MPN/mL.

### Statistical Analysis

Statistical tests were conducted in RStudio version 4.4.1. Normality was evaluated with the Shapiro–Wilk test, and equal variance was checked using the Bartlett and Levene’s tests. Linear models were fit for the total chlorine concentration in the water heater versus *Lp* or *Ma* leaving the water heater. Modeled versus approximate area CT values were compared with a paired Wilcoxon rank-sum test. Significance was defined at *p* < 0.05 for all tests.

## Results and Discussion

### Disinfectant Decayed Quicker in Plumbing Than Glass Jars

The rate of chloramine decay was dramatically higher in pipes compared to batch solutions in glass jars, which represent idealized plug flow decay kinetics without pipe wall or biofilm effects.^33^ The estimated time required for chloramine to decay from 1 to 0.05 mg/L at 25 °C ranked as follows: tap water in a glass jar (≈6880 h) ≫ GAC-filtered water in a glass jar (≈580 h) ≫ PEX cold water pipes with biofilm (3.7–13 h). In the glass jars, the time required for chloramine to decay from 1 to 0.05 mg/L was about 2–3 orders of magnitude longer than in the presence of PEX pipe colonized by mature biofilms. Even when the water in the glass jars with GAC inoculum (182 total cells/mL) had a comparable starting total cell count to the pipe rig influent, the chloramine persisted 93× longer in the jars than in the pipes.

### Disinfectant Decay and Penetration into the Rig

All of the hot water taps and cold water taps with >1 day WRT were found to have disinfectant levels below 0.15 mg/L after normal daily flushing (Figure S3), likely as a result of nitrification.^33^ Consequently, chloramine decay profiles could only be monitored at the 1 day WRT cold water taps. Disinfectant decay profiles were similar at all cold 1 day WRT taps after two months when the feedwater contained 1 mg/L chlorine residual (Figure 2a). These pipes were exposed to chloramine once per day with each pipe flush during normal operations, though a 2–3× pipe volume flush was needed while monitoring decay to reach chloramine levels comparable to the rig influent. As influent disinfectant residual and contact time increased, some substantial differences in chloramine decay profiles were observed among different taps dependent on flow rate and pipe diameter. For instance, three and a half months after the influent residual was elevated to ∼2.5 mg/L, decay profiles for the duplicate high flow rate 8.3 L/min pipes, and the 19.05 mm diameter low flow rate pipe had a more persistent chloramine residual than the other conditions (Figure 2b). An increased persistence of chloramine was observed in these three pipes following a 6.5 month period, during which the influent was maintained at ∼2.5 mg/L (Figure 2c). We speculate that a lower surface-area-to-volume ratio was one factor that impacted the slower decay rate of the 19.05 mm pipe compared to other 1 day WRT pipes of the same flow rate.^33,34^ Transient high-velocity flow has also been reported to reduce disinfectant demand in distribution system pipes or biofilm reactors by preventing particle accumulation or shearing loose biofilm that accumulates during stagnation.^35–42^ Such factors might explain the slower decay in the high-velocity 8.3 L/min pipes. In this study, the pipes were stagnant 99.96% of the time, and all had the same WRT. For buildings with higher or irregular water use, it is expected that differences in shear velocity and water age between taps would influence disinfectant demand.^40^

**Figure 2:**
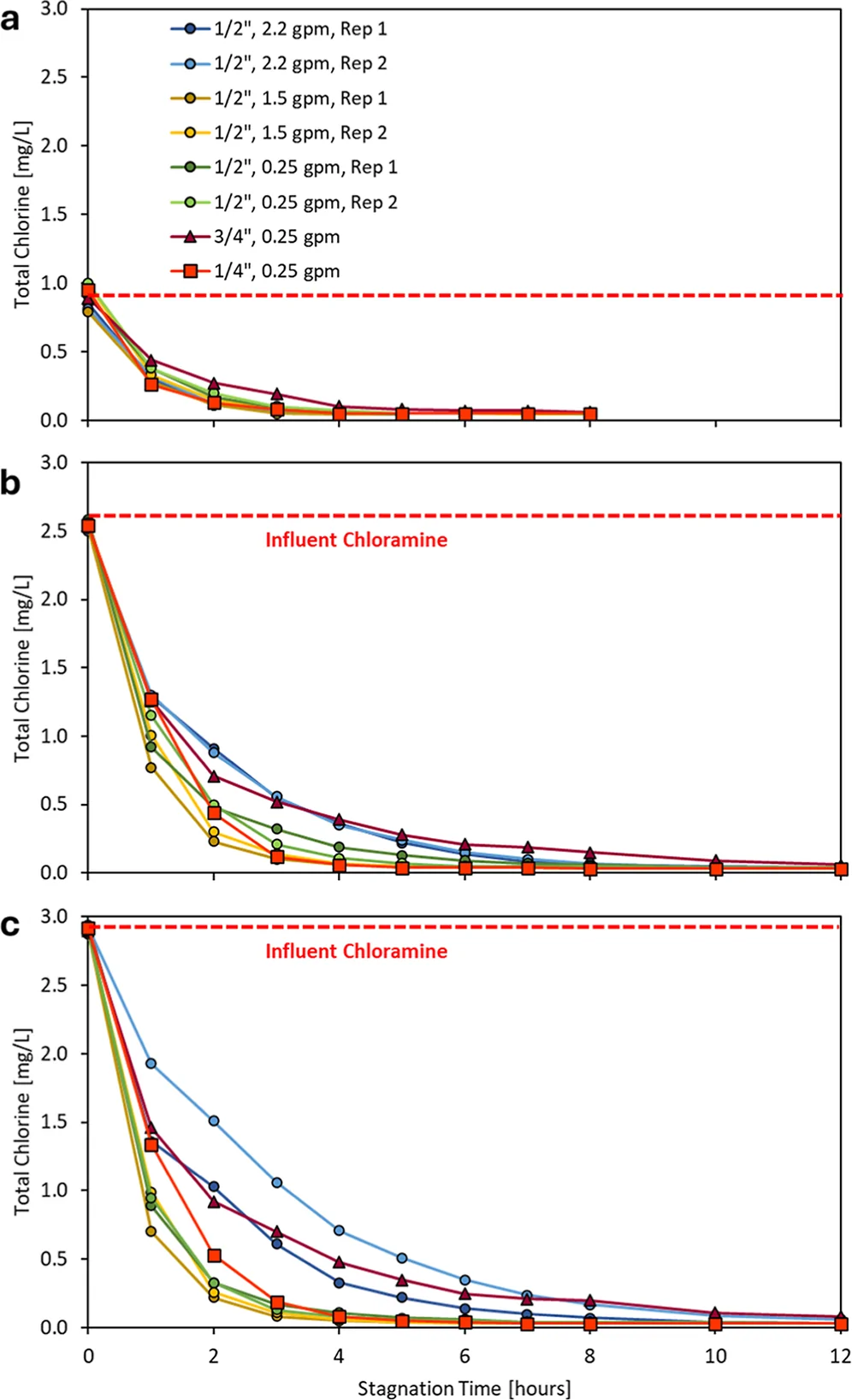
Representative chloramine decay profiles at cold 1 day WRT taps. Influent chloramine concentrations were (a) 1.0 mg/L for 2 months, (b) ∼2.5 mg/L for 3.5 months, and (c) ∼2.5 mg/L for 6.5 months. Decay profiles are shown for 8 taps, with each tap being located on a separate PEX pipe at a room temperature of 25 °C. Pipe diameters ranged from 6.35 to 19.05 mm, and flow rates varied from 0.95 to 8.3 L/min. The 12.7 mm pipes are duplicated. The dashed red line references the influent chloramine concentration entering the pipes during the daily 35 s flush. The chloramine level entering the rig was abnormally high near 3 mg/L on the day of the final decay profile evaluation.

Even though all taps were flushed daily for 35 s, designed to deliver the exact volume necessary to displace the water in the pipe for a 1 day WRT, the initial chloramine concentrations were not identical across the taps immediately following the flush. Chloramine concentrations were measured at all taps before and after the daily flush at time points of 3, 13, and 23 weeks following the transition to the highest target daily influent chloramine level of ∼2.5 mg/L (Figure S3). During normal daily operations, the chloramine concentrations at the cold 1 day WRT taps never reached levels measured at the influent and, in one case, were <5% of the influent concentration following 3 weeks of flushing (Figure S3a). This is beyond the chloramine deficit that can be reasonably accounted for by nonideal plug flow in the pipes. With operation for 13 weeks at an influent chloramine at ∼2.5 mg/L, there was an upward trend in the initial residual at cold 1 day WRT taps, with the increased contact time at the high chloramine dose (Figure S3b). This demonstrated that, though the chloramine level entering the rig was constant, residual at the tap directly following flushing rose in portions of the pipes over time as the system adapted to the higher disinfectant dose. We expected that the chloramine concentrations at all taps directly following flushing would continue to increase, but three additional months of exposure to full-strength chloramine did not result in postflush residual concentrations at the taps increasing or in substantial disinfectant penetrating past the 1 day WRT in cold or hot pipes (Figure S3c). Other studies have reported rapid bacterial regrowth and increased biofilm density under chlorinated, nutrient-available conditions.^43,44^ Such conditions were likely also at play in the at-scale plumbing rig, effectively diminishing the PPCT in this particular instance.

### PPCT in the Rig Pipes

Chloramine was undetectable at all the 1 day WRT taps across the rig after either 8 or 24 h of stagnation. Our measurement of chloramine over multiple time points during stagnation revealed differences in CT at each of the cold 1 day WRT taps. At an influent chloramine level of 0.5 mg/L, CT varied from 32 to 40 mg min/L (Table 1). Increasing the influent chloramine level to 1 and ∼2.5 mg/L produced proportionate increases in CT across all taps. For instance, the average CT value increased by 2.05× at the cold 1 day WRT taps when chloramine doubled from 0.5 to 1 mg/L and by 6.21× when chloramine increased from 0.5 to ∼2.5 mg/L.

**Table 1:** Chloramine PPCT Values Measured at Cold 1 Day WRT Taps at a 25 °C Room Temperature

	CT (mg min/L) in Cold 1 Day WRT Taps at Indicated Target Influent Cl_2_			
Pipe Diameter (mm), Flow Rate (L/min), Replicate^a^	0.5 mg/L (1.5 Months)	1.0 mg/L (2 Months)	2.5 mg/L (3.5 Months)	2.5 mg/L (6.5 Months)^b^
12.7, 8.3, R1	34.8	65.4	289.8	276.1
12.7, 8.3, R2	39.3	66.0	292.5	424.0
12.7, 5.7, R1	32.1	60.6	153.9	131.5
12.7, 5.7, R2	39.9	77.1	177.6	149.2
12.7, 0.95, R1	38.1	77.7	211.2	159.2
12.7, 0.95, R2	37.2	85.2	206.7	157.4
19.05, 0.95	NM^c^	101.7	308.7	344.4
6.35, 0.95	NM^c^	70.2	197.7	184.7
Average ± Std Dev	36.9 ± 2.7^d^	75.5 ± 12.4	229.8 ± 54.9	228.3 ± 100.9
RSD^e^	7.3%^d^	16.4%	23.9%	44.2%

a The ability to replicate pipe conditions was limited by the plumbing manifold ports.

b Due to fluctuations in the disinfectant residual of the town water supply, the influent chloramine concentration was 2.93 mg/L on this day of sample collection. The starting residual was adjusted to 2.5 mg/L to remove the impact of this abnormally high influent chloramine concentration.

c NM = not measured.

d Excludes the 6.35 and 19.05 mm pipes.

e RSD = relative standard deviation.

No previous studies were identified that describe how disinfectant decay and CT respond to prolonged exposure to elevated chloramine concentrations. We hypothesized that if sustained chloramine exposure progressively inactivated microorganisms that cause disinfectant decay (e.g., nitrifying bacteria), chloramine persistence would increase. Conversely, if the pipe biofilm acclimated or developed resistance to the disinfectant, then nitrification could be enhanced along with accelerated chloramine decay.^9,23,30^

We found that the average CT values at ∼2.5 mg/L influent levels remained relatively constant at 229.8 mg min/L at 3.5 months compared to 228.3 mg min/L at 6.5 months (Table 1). In terms of conditions that changed after three additional months of high chloramine, two of the conditions increased in CT by 11.6–45.0%, suggesting that the chloramine was gradually weakening biofilm-associated decay for those cases, whereas CT in the remaining six conditions decreased by 4.7–24.6%, indicating that the biofilms were developing increased resistance to disinfection.

Disinfectant concentration was a factor that affected the consistency of residual stability. The relative standard deviation for CT among all 1 day WRT pipes progressively increased from 7.3% at 0.5 mg/L influent chloramine to 44.2% in the ∼2.5 mg/L condition measured at 6.5 months, indicating that increased variability in decay profiles could be associated with higher influent residuals. Some variability was even observed between duplicate conditions, such as the 12.7 mm 8.3 L/min pipes, where CT differed by 147.9 mg min/L. This further demonstrates the unpredictability of decay trends in premise plumbing as a result of the corresponding microbiomes and their response to disinfection. Field studies have detected differences in microbial community structure in both new and old premise plumbing systems even in sections just 1 m apart.^45,46^

CT values derived from glass jars simulating bulk water reactions without plumbing materials or biofilm were confirmed to be a poor surrogate for PPCT. The reaction *n* and *k* values reported by Odimayomi et al.^33^ highlight the major difference in chloramine decay kinetics between the glass jars (*n* = 0.32, *k* = 90.1 L^*n*–1^/(mg^*n*–1^ h)) and cold 1 day WRT pipes (*n* = 1.18–2.74, *k* = 0.37–34.35 L^*n*–1^/(mg^*n*–1^ h)) modeled using the linear integrated rate law.^33^ Receiving the same tap water at ∼25 °C with nitrifiers and a 2.5 mg/L initial chloramine residual, the CT in glass jars was 1123 ± 3.5 mg min/L, while the corresponding CT in the pipes was 5× lower at 229 ± 81 mg min/L. Extending to a 24 h period, CT was 12× lower in the pipes versus the glass jars.

### PPCT Concept in Water Heaters

In a stagnant distal pipe segment of reasonably short length, the chloramine concentration throughout the entire pipe is expected to be uniform, assuming that all of the water in the pipe section was displaced during flushing. This makes the calculation of CT during a stagnation event near a distal tap relatively straightforward. However, certain sections of a premise plumbing system are more heterogeneous. In such sections of plumbing, only a portion of the total water volume is displaced during a flushing event, as is the case for a water heater. For example, as water enters a water heater, it may have roughly plug-flow behavior and only displace water at the bottom of the tank, leaving water at higher sections of the tank relatively undisturbed. Alternatively, the tank could have nearly completely mixed behavior similar to that of a continuous stirred tank reactor, especially if the system had a recirculating pump, causing new water entering the tank to mix with water of an older water age.

Given such complexities, the actual levels of chloramine throughout a water heater tank during days or weeks of stagnation are difficult to predict. In this study, we estimated PPCT in the tank based on the chloramine decay profile constructed from water samples collected at the top of the water heater through the anode rod port (see assumptions in Figure S1 and eq S1). After the daily flush introduced 18.6 L (i.e., 25.8% of the tank volume) of influent water at ∼2.5 mg/L chloramine into the tank, activation of the single heating element triggered convective mixing, which increased the disinfectant concentration at the top of the tank from trace levels to 0.52 mg/L within minutes. This concentration approaches the 0.65 mg/L predicted for complete mixing of the one-quarter influent-to-existing tank volume ratio. Chloramine then decayed to 0.11 mg/L at the top of the tank following approximate first-order decay kinetics over 24 h (Figure S4). Thus, at an influent residual of ∼2.5 mg/L, the water heater tank was estimated to have a 24 h PPCT of 342.9 mg min/L.

### *L. pneumophila* Growth in the Rig as a Function of PPCT

The calculated PPCT at each of the cold 1 day WRT taps was considered relative to the concentration of culturable *Lp* measured at those taps as the influent chloramine increased. The likelihood of *Lp* growth at cold taps with a 1 day WRT had a striking dependency on PPCT (Figure 3).

**Figure 3:**
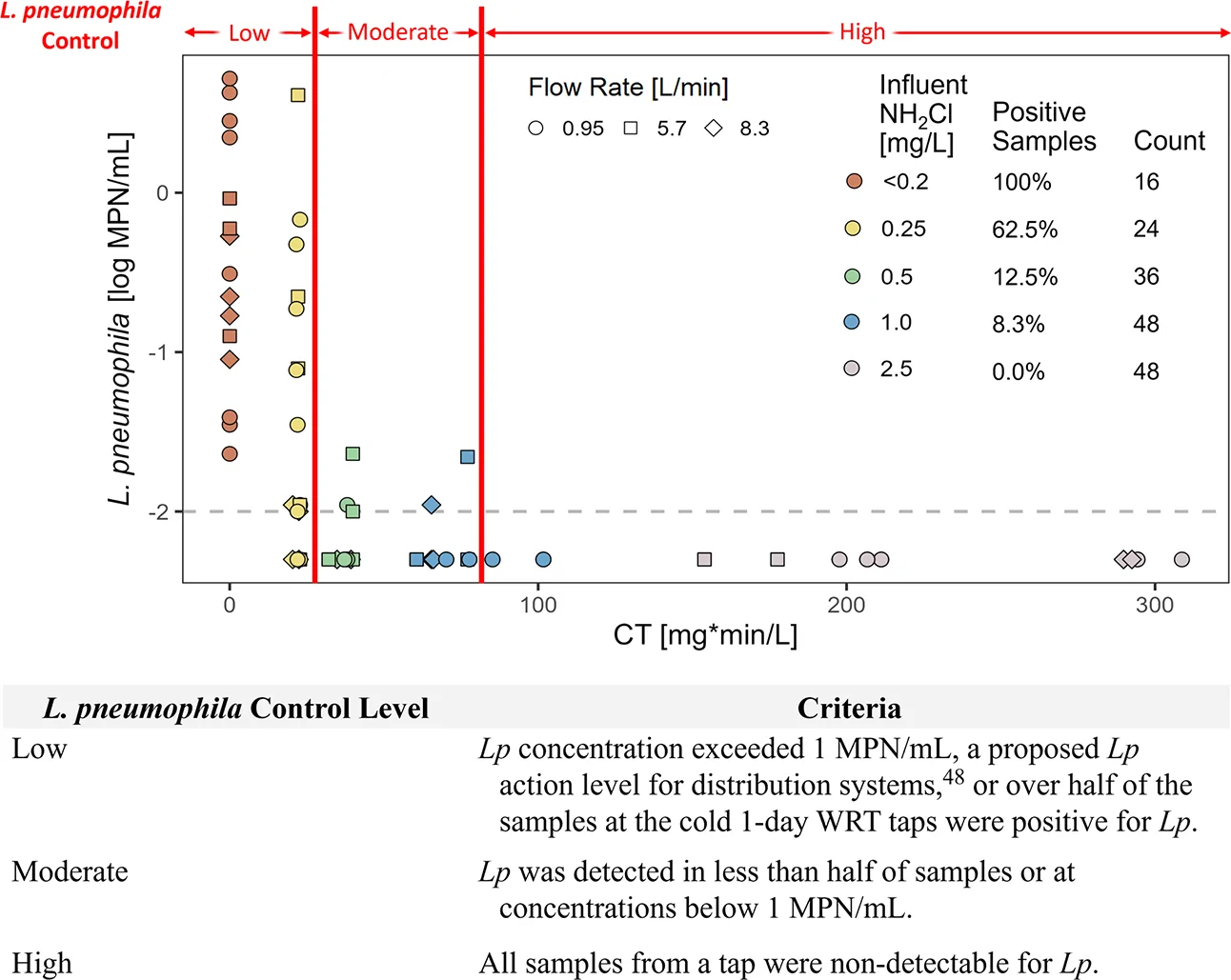
Effect of PPCT on culturable *L. pneumophila* concentration at cold 1 day WRT taps. Colors represent increasing influent chloramine. Shapes represent flow rate. The room temperature was consistent at 25 °C. Solid red lines separations denote the cutoffs applied in this study defining low, moderate, and high control of *L. pneumophila*.^48^ The gray dashed line represents the detection limit of 0.01 MPN/mL. Nondetectable results were set equal to half the detection limit.

### *L. pneumophila* Control Thresholds

For further evaluation of the data and corresponding trends, we defined three levels of control for *Lp* in the cold 1 day WRT taps: low, moderate, and high controls (Figure 3). Low control occurred at PPCT values between 0 and 25 mg min/L as 100% of the cold 1 day WRT taps tested positive for *Lp* with a trace influent chloramine concentration, which decreased to 62.5% of samples with detectable *Lp* at 0.25 mg/L chloramine (Figure 3). Moderate *Lp* control was achieved with PPCT values between 25 and 78 mg min/L. Within this range, 11% of samples were positive for *Lp* when the influent chloramine was set to 0.5 or 1.0 mg/L. At PPCT values above 78 mg min/L, no samples yielded detectable *Lp*, consistent with high control. All hot water taps and cold taps with >1 day WRT in this study received no disinfectant residual and had 100% positivity for *Lp*, indicating low control.^47^

### Room Temperature, a Prerequisite for Growth

All of the above-mentioned *Lp* detections for cold water taps were observed at a relatively warm room temperature of 25 °C. *Lp* was never detected in the cold water pipes when operated at a room temperature of 18 °C, prior to increasing to 25 °C in the experiments summarized herein. This is consistent with prior research indicating that 18 °C is within the dormant or very slow growth range of *Legionella* activity.^49^ Thus, at a sufficiently low ambient temperature, a minimum PPCT is not essential to control growth in cold water pipes.

### Comparison of PPCT and Published CT Values

The recurring daily disinfection PPCT of >78 mg min/L that resulted in nondetectable levels of *Lp* at the cold 1 day WRT taps is in close agreement with the Buse et al. reported^17^ one-time disinfectant dose of 62.8 mg min/L needed for 3-log inactivation of *Lp* on biofilm reactor PVC slides in an inert glass beaker (Table 2). For comparison, Dupuy and colleagues found that the chloramine CT required to achieve 3-log inactivation was 16 mg min/L for *Lp* alone and 18–22 mg min/L for *Lp* cocultured with *Acanthamoeba* spp.^50^ In buildings with no known history of *Lp* colonization and in areas of plumbing that cannot effectively shelter *Lp*, PPCT could function as a strategy to suppress growth instead of targeting disinfection.

**Table 2:** Published CT Values for 3-log10 Inactivation of *L. pneumophila* or *M. avium* from Chloramine Treatment

Study	Disinfection Temperature (°C)	Solution	Target	Acclimation to Solution Before Disinfection	Mode	CT Value (mg min/L)
Present study^a^	25	Drinking water	*L. pneumophila* wild strain	In site	Water collected directly from PEX pipe with biofilms aged 4–6 years	78
Buse et al., 2019 ^17^	21–23	Sterile drinking water	*L. pneumophila* Philadelphia-1	None	Planktonic in conical tubes	6.6
			*L. pneumophila* Philadelphia-1	5 day incubation with biofilm coupons	PVC coupon biofilms aged for months^b^ in annular reactor before transferring to inert glass beaker	62.8
			*L. pneumophila* Philadelphia-1	5 day incubation with biofilm coupons	copper coupon biofilms aged for months^b^ in annular reactor before transferring to inert glass beaker	55.4
Dupuy et al., 2011 ^50^	30	Sterile phosphate buffer	*L. pneumophila* Lens	None	Planktonic in inert glass container	16
			*L. pneumophila* Lens cocultured with *Acanthamoeba* spp.	None	Planktonic in inert glass container	18–22
Taylor et al., 2000 ^26^	23	Phosphate buffer	Monochloramine susceptible *M. avium* strains A5 and 5502	NR^c^	Planktonic in stoppered inert glass Erlenmeyer flask	91–97
			Monochloramine resistant *M. avium* strains 1060, 1508, and 5002	NR^c^	Planktonic in stoppered inert glass Erlenmeyer flask	458–1710

a *L. pneumophila* reduction in the present study was at least 3-log10.

b The precise duration of biofilm growth was not specified.

c NR, not reported.

### *L. pneumophila* in the Water Heater

The PPCT thresholds for *Lp* at 25 °C room temperature in PEX pipes were distinct from those determined for the water heater tank at 40 °C with approximate complete mix conditions. As the influent chloramine dose increased, the estimated CT in the tank (calculated based on eq S1) increased up to 342.9 mg min/L and *Lp* concentration significantly decreased in the water heater (*n* = 15, *p*-value = < 0.001) (Figure S5). Despite this high CT, *Lp* persisted in the water heater effluent at 1.03 log MPN/mL, demonstrating that disinfectant alone is not a silver bullet for OPs (Figure S6). Thus, a multibarrier approach is sometimes needed to control OPs in drinking water distribution systems and premise plumbing. Unlike the pipes, we hypothesize that sediment at the bottom of the tank harbored and sheltered *Lp* from chloramine, requiring the PPCT to function as a mechanism for killing microbes rather than just preventing colonization.

Sediments in drinking water storage tanks can act as a reservoir for OPs.^28,29^ Investigations indicated that the tank in this study contained a white sediment that was ∼10 cm deep and enriched in *Lp* at a concentration of 2.26 log MPN/mL, even after 10 months at an influent chloramine concentration of ∼2.5 mg/L. Thus, it is hypothetically possible that the detection of *Lp* leaving the tank was due to tank sediment containing high *Lp* being stirred up at the onset of flow during the daily flush as inflowing cold water displaced water at the bottom of the tank. Additionally, the heating element in the tank turned on after flushing and at approximately 10 h intervals, likely causing convective mixing to resuspend sediment.^51^ Such hydraulic disturbances could result in *Lp* short-circuiting—*Lp* containing sediment from the tank bottom, where there is potentially no chloramine, exiting the tank effluent outlet in a matter of seconds to minutes.^52,53^ As is the case for calculating CT of sedimentation basins, tracer studies of water heaters along with observation of sediment disturbances are needed to explain the persistence of *Lp* in the water heater effluent. The importance of sediment source (e.g., debris from the distribution system or corroded anode rod deposits) also warrants further investigation.^25^

### Enhanced *M. avium* Growth in the Water Heater

There was a stark difference between the response of *Ma* to increasing chloramine in the rig compared to *Lp*. If short-circuiting of sediment did not occur, the *Ma* strain that naturally colonized the rig in this study not only withstood a PPCT up to 342.9 mg min/L in the water heater but also significantly increased to a concentration of 4 log colony-forming units (CFU)/mL in the water heater as the influent chloramine level rose to ∼2.5 mg/L (*n* = 5, *p*-value = 0.037) (Figure S5). *Ma* is extremely resistant to chlorine disinfectants,^26,54,55^ and *Mycobacterium* spp. detection has been reported to increase in a water distribution system after switching from chlorine to monochloramine as the secondary disinfectant.^56^ A study of *Ma* disinfection found that, when grown in Middlebrook 7H9 broth and treated at 23 °C, three of the five strains tested were resistant to monochloramine and had 3-log inactivation CT values between 458 and 1710 mg min/L (Table 2).^26^ Surprisingly, the amount of *Ma* leaving the water heater did not decrease at all even though the PPCT was near this lower threshold for 3-log inactivation of planktonic cells. Beyond *Ma* disinfection resistance, future research should consider the role of sediment and microbial competition on *Ma* growth in chloraminated water systems. Regarding other locations in the rig, *Ma* quickly died off in the cold pipes at >0.2 mg/L chloramine and generally decreased with increasing water age in the hot pipes compared to levels measured in the water heater (data not shown). We hypothesize that the inability for *Ma* to grow outside of the water heater was a result of the pipes being held at a room temperature below the reported *Ma* growth range of 28–50 °C.^57–59^ CT theory assumes that the disinfectant does not support the growth of the target organism. However, for *Mycobacterium avium* complex in chloraminated systems, further investigation is needed to identify the concentration at which chloramine begins contributing to PPCT.

### Modeling CT in Premise Plumbing

In a previous study, we developed best-fit models that can be applied for a premise plumbing disinfectant decay curve.^33^ Here, we explored whether best-fit decay models could effectively predict PPCTs (i.e., the area under the disinfectant decay curve). However, the fit of the models was highly dependent on the modeling method and the reaction order and rate coefficients used. For example, for cold 1 day WRT taps, the best fit nonlinear least-squares method was able to accurately fit the results of the control method (i.e., approximated area CT values from Figure S1 and eq S1) underestimating PPCT by only 5% on average with an error range of −17 to 14% (paired Wilcoxon test, *p*-value = 0.115) (Table S2). On the other hand, the best-fit linear integrated rate law method did not fit PPCT measurements; as a result, it underestimated PPCT by an average of 33% with error ranging from −86 to 17% (paired Wilcoxon test, *p*-value = < 0.001). Simply assuming a reaction rate coefficient or an integer reaction order can further increase error when fitting disinfectant decay and, therefore, PPCT.^33^ Further, the disinfectant decay rate at a tap should not be assumed to remain constant. Accurate estimation of PPCT likely requires characterization of the tap-specific disinfectant decay profile, which is influenced by plumbing age, usage patterns, water chemistry, seasonal variability, and location within the building. PPCT should be measured at the tap periodically, as these factors change.

## Conclusions

This at-scale pipe rig study demonstrates how the traditional CT concept can be adapted to conditions encountered in premise plumbing (PPCT), informing frameworks for OP control. A similar concept was recently implemented by the AWWA standards committee to monitor CT as a tool for disinfecting newly constructed or renovated buildings for a one-time treatment to achieve at least 3-log inactivation of bacterial and viral pathogens that may be introduced during construction.^60^ Such short-term interventions, however, when applied as a one-time treatment toward in-building OP control without appropriate management afterward, allow potential regrowth following treatment.^43,61^ Whereas OPs can be repressed with a sufficient routine PPCT or other approaches. The PPCT could also help to assess whether a building’s normal operation or flushing protocol is capable of continuously suppressing OP growth or to guide selection of target doses for in-building disinfection when the conventional disinfectant residual is insufficient.

Flushing programs could also be implemented to maintain minimal PPCT targets at the distal taps. In our case, the pipes were flushed on a routine basis with a predictable influent residual. The daily PPCT could be increased by raising influent chloramine levels or by more frequent flushing events. For instance, assuming that the chloramine decay kinetics in the pipes were unchanged, implementing two daily flushing events 12 h apart would have doubled CT in our test rig, or three daily flushing events 8 h apart would triple CT. Such a linear relationship between flushing events and daily PPCT will occur as long as the residual decays to zero before the next flushing event. At another extreme, our approach illustrates how repeated flushing would have almost no impact on PPCT if there is no residual in the influent or in waters where chlorine decay is insignificant over a 24 h period, as we observed in our glass jars. Future research would need to establish minimum PPCTs that are considerate of fluctuations in the weekly flow patterns, influent residual, and residual decay rates in a building.

CT was developed for enteric pathogens that do not grow in water and is not generally applied to small pipes with biofilms, yet PPCT within pipes still explained trends in *Lp* occurrence despite chloramine decay rates in premise plumbing being 2–3 orders of magnitude greater than those in glass jars. Low PPCT associated with chloramine doses below 0.5 mg/L did not effectively suppress the growth of *Lp* at cold 1 day WRT the taps. An influent chloramine dose corresponding to CT values below 25 mg min/L represented a threshold PPCT for moderate *Lp* control in this system. *Lp* was subsequently detected sporadically until the daily PPCT reached 78 mg min/L, at which point *Lp* was undetectable.

Similarly, the PPCT monitoring technique presented here can be used to identify a threshold for control in buildings, though disinfectant type and plumbing materials should be considered when setting PPCT requirements. For example, Buse et al. reported that the CT required for a 3-log inactivation of biofilm-associated *Lp* was 26.7 mg min/L higher for monochloramine compared to free chlorine on PVC coupons, whereas the corresponding CT difference on copper coupons was only 4.6 mg min/L.^17^ PPCT will likely have temporal and locational variability within a building during normal weekly use as observed in the present study (Table 1). Of the taps that had a detectable residual, PPCT values varied from 131.5 to 424.0 mg min/L (44.2% RSD) after 6.5 months of operation at the highest influent chloramine concentration. Temperature stood as a prerequisite for pathogen growth, with *Lp* and *Ma* only colonizing and proliferating in portions of the rig within the temperature range suitable for growth.

The method of measuring PPCT in buildings developed and applied in this study is expected to be well-suited to monitoring residuals inside the pipes. Determining the disinfectant decay profile at a location that does not have a tap (e.g., a point along the length of a pipe) would require large volumes to be collected and replenished during sampling, thereby creating inconsistencies. It is also preferable to monitor at a distal tap far from a branching point to ensure that water use in other portions of the building does not interfere with the measurements. Accordingly, monitoring should be conducted at locations where flow is completely controlled by the sampler during the collection of a decay profile (e.g., roughly 8 h herein). CT monitoring should ideally be conducted at different locations in a building and after notable changes in the water temperature, disinfectant dose, or disinfectant type.

Disinfectant decay models, such as the best-fit models used herein, can be useful for estimating PPCT. However, such models are sometimes applied to predict trends in disinfectant residuals using default reaction parameters without system-specific measurements. Empirical data are essential for reducing error, as measured reaction order and rate coefficients differ substantially from those in default models and can even vary considerably from tap to tap within a given building. When properly measured or predicted, PPCT may provide a useful measure to ensure disinfectant control of opportunistic pathogens in premise plumbing and explain trends in OP occurrence.

## Supplementary Material



## Notes

Illustration of Premise Plumbing Disinfectant Concentration × Stagnation Time (PPCT) calculation for chloramine decay curves; exemplary PPCT calculation for the longest-lasting residual in this study; time needed to reach a 0.1 mg/l total chlorine residual at 1 day WRT taps during decay profiling; PPCT at the tap; disinfectant decay in glass jars; Linearized Integrated Rate Law (LIRL) chloramine decay model; Nonlinear Least Squares (NLS) chloramine decay model; chloramine residual before and after flush; water heater disinfectant profile; water heater pathogen trends as a function of chloramine concentration; water heater pathogen and total cell trends with PPCT; and modeled Best Fit *n*th order chloramine PPCT percent error from measured PPCT (PDF)

## §

Black and Veatch, 11401 Lamar Ave, Overland Park, Kansas 66211, United States
