# Supplementary material for: Premise Plumbing “CT” (PPCT) as a Control Parameter for Opportunistic Pathogens
Source: ACS ES T Water. 2026 Jul 23;6(8):5398–407. doi: 10.1021/acsestwater.6c00582 (PMC13488392; doi:10.1021/acsestwater.6c00582)
Supplement: Supplementary file 1 [file ew6c00582_si_001.pdf]

Supplemental Information for

PREMISE PLUMBING "CT" (PPCT) AS A CONTROL PARAMETER FOR OPPORTUNISTIC PATHOGENS

Tolulope O. Odimayomi[1]*, Darel C. Snead[1], William J. Rhoads[1,2], Myra D. Williams[3], Joseph O. Falkinham III[3], Amy Pruden[1], and Marc A. Edwards[1]

[1]Via Department of Civil and Environmental Engineering, Virginia Tech, Blacksburg, Virginia 24061, United States

[2]Current affiliation: Black and Veatch, 11401 Lamar Ave, Overland Park, Kansas 66211, United States

[3]Department of Biological Sciences, Virginia Tech, Blacksburg, Virginia 24061, United States

*Email: todimayo@vt.edu

SI 1. Acclimation of the Rig and Colonization of Pathogens

Initial investigation showed that the rig was naturally colonized with a wild strain of *Mycobacterium avium*. *Legionella pneumophila* (strain isolate from Quincy, IL)[1] was inoculated into the 72 L water heater through the anode rod port at a concentration of ~4,000 most probable number (MPN)/L from another plumbing rig. The inoculum in the water heater was mixed before being distributed to hot water pipes via the normal flow path and cold pipes via a diverted flow path. Inoculation took place in all pipes at the start of the experiment at a room temperature of 18°C and was repeated in cold pipes after the room temperature was increased to 25°C. Following each inoculation, water containing the inoculum was held in the pipes for 24 h before normal flushing was resumed.

SI 2. Water Heater Sediments

After the rig received an influent chloramine dose of ~2.5 mg/L for 10 months, 2 liters of water containing sediments was collected from the water heater drain valve into a sterile polypropylene bottle. Culturable *L. pneumophila* in the sediment solution was measured at a 1:100 dilution with autoclaved rig influent water as the diluent.

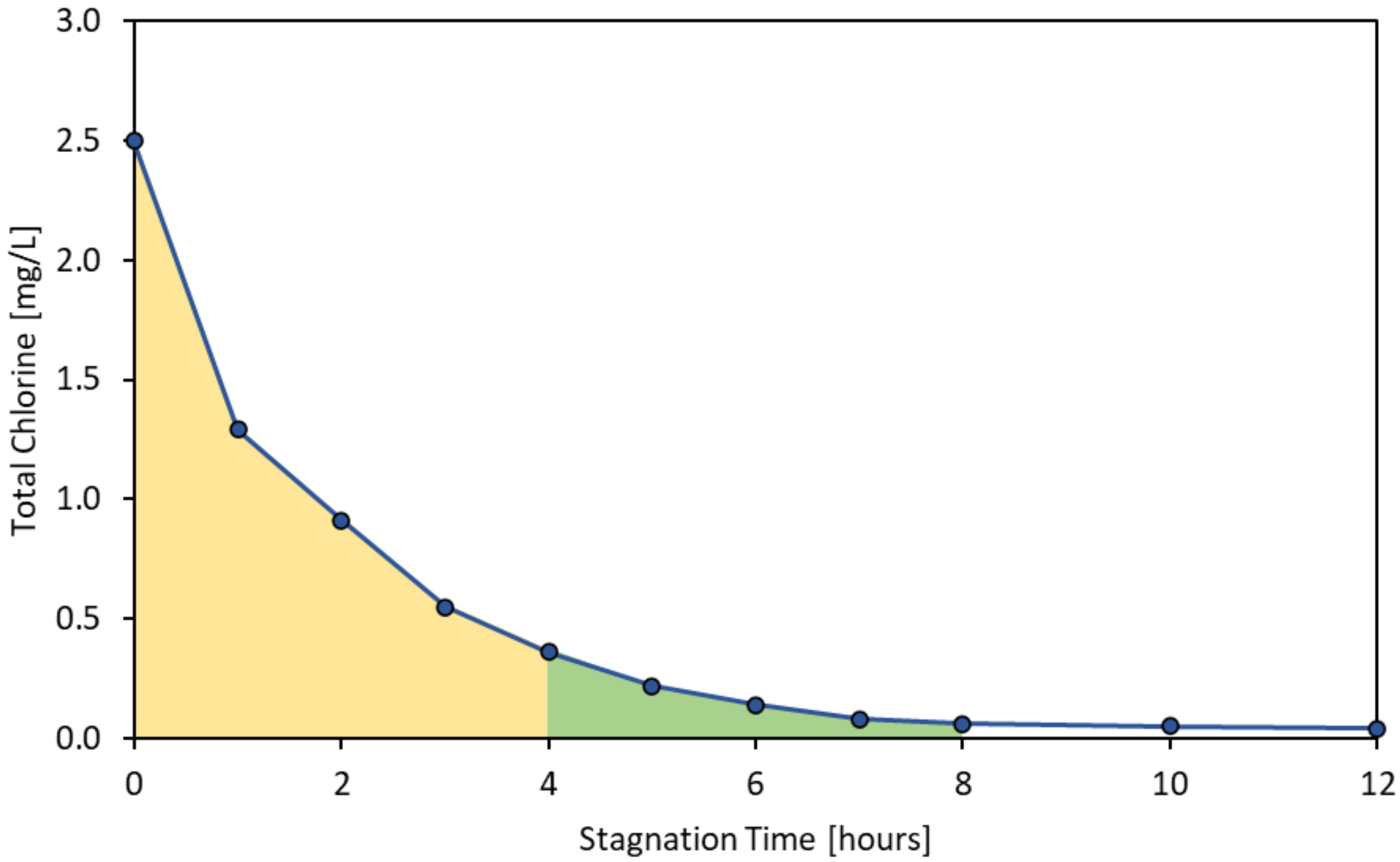

Figure S1. Illustration of Premise Plumbing Disinfectant Concentration × Stagnation Time (PPCT) Calculation for Chloramine Decay Curves. Yellow shaded area: CT for time 0-4 hours. Green shaded area: CT for time 4-8 hours. The 8-hour CT is the sum of both the yellow and green shaded areas.

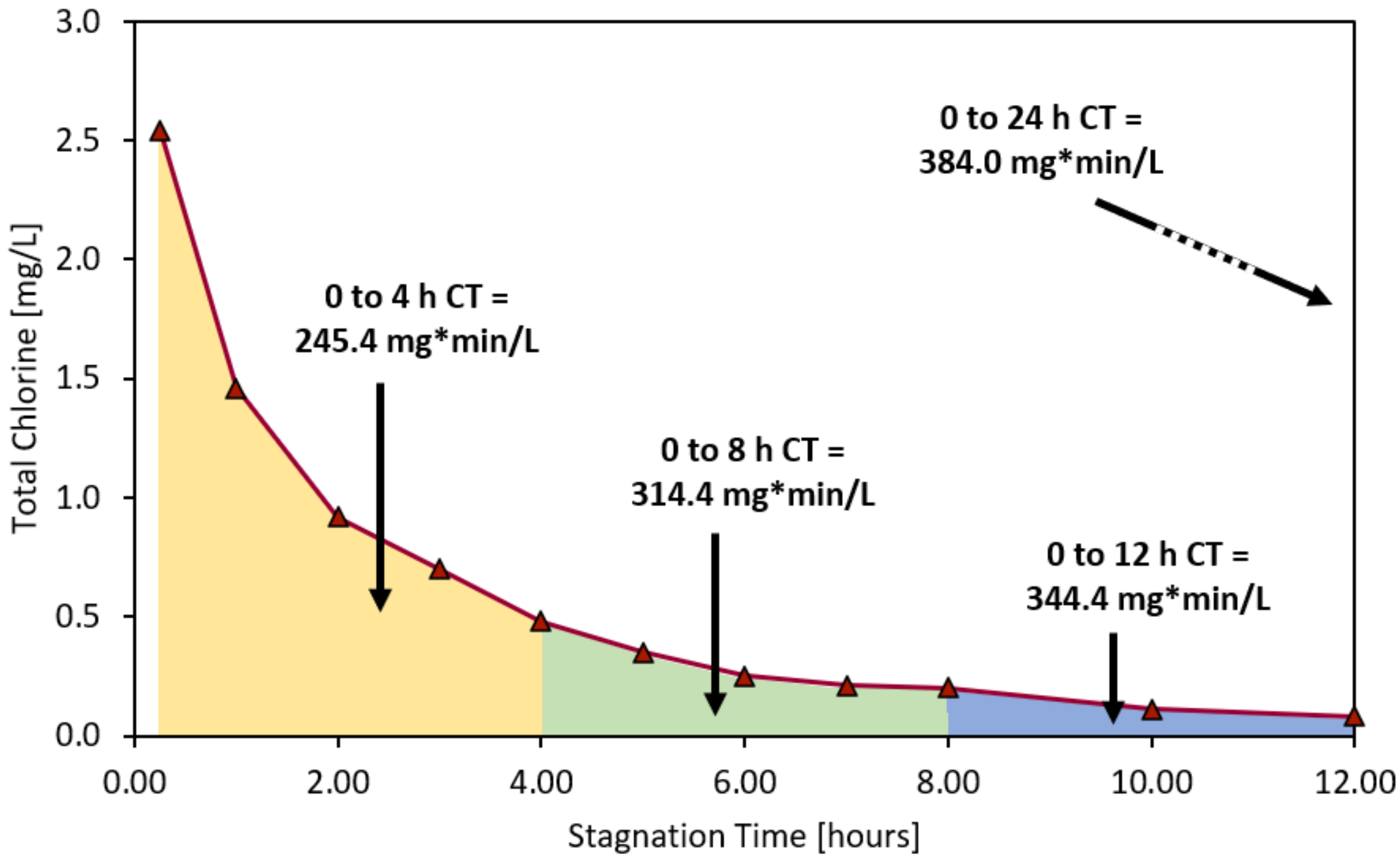

Figure S2. Exemplary PPCT Calculation for the Longest Lasting Residual in this Study. The 19.05 mm, 0.95 L/min cold 1-day WRT tap has a straightforward integration of 344 mg*min/L for the first 12 hours. But the total chlorine measurement at 12 hours was 0.08 mg/L and at 24 hours it was 0.04 mg/L. If it is assumed that these low values are accurate representations of chloramine, the integrated area from 12-24 hours would 43.2 mg*min/L, representing a 13% increase from the PPCT measured from the start up to 12 h. On the other hand, if this trace level of chlorine was an artifact at 0.08 mg/L and below, due to false positivity from organochlorine, the additional CT from 12 to 24 hours would be zero. For this study, we used a convention that did not include the incremental CT from total chlorine below 0.1 mg/L after 12 h in PPCT calculations.

Table S1. Time Needed to Reach a 0.1 mg/L Total Chlorine Residual at 1-Day WRT Taps During Decay Profiling

Pipe Diameter (mm), Flow Rate (L/min), Replicate[a]	Time to Reach a Residual ≤ 0.1 mg/L [hrs]			
	Target Influent Cl_2 = 0.5 mg/L (1.5 Months)	Target Influent Cl_2 = 1.0 mg/L (2 Months)	Target Influent Cl_2 = 2.5 mg/L (3.5 Months)	Target Influent Cl_2 = 2.5 mg/L (6.5 Months)[b]
12.7, 8.3, R1	2	3	7	8
12.7, 8.3, R2	2	3	7	10
12.7, 5.7, R1	2	3	3	3
12.7, 5.7, R2	2	3	4	4
12.7, 0.95, R1	2	3	6	5
12.7, 0.95, R2	2	3	5	4
19.05, 0.95	NM[c]	4	10	12
6.35, 0.95	NM	3	4	4

[a]The ability to replicate pipe conditions was limited by plumbing manifold ports.
[b]Due to fluctuations in the disinfectant residual of the town water supply, the influent chloramine concentration was 2.93 mg/L on this day of sample collection. The starting residual was adjusted to 2.5 mg/L to remove the impact of this abnormally high influent chloramine concentration.
[c]NM = not measured

Equation S1. PPCT at the Tap

n^{th}-hour CT = ∑ (

(1-hour total chlorine * (1-hour – 0-hour))

+ 0.5 * ((0-hour total chlorine – 1-hour total chlorine) * (1-hour – 0-hour))

…

(n^{th}-hour total chlorine * (n^{th}-hour – (n^{th}–1)-hour))

+ 0.5 * (((n^{th}–1)-hour total chlorine – n^{th}-hour total chlorine) * (n^{th}-hour – (n^{th}–1)-hour))

)

SI 3. Modeling PPCT

In a situation with relatively infrequent flow events, we introduce PPCT as the area under the chloramine decay curve in a pipe during stagnation, which was estimated by taking the double integral of the best-fit decay model:

$$\frac{dC}{dt} = -kC^n \qquad (1)$$

where C is chlorine concentration at time t (mg/L), n is the reaction order, and k is the chlorine reaction rate coefficient (variable units dependent on n). The method of measuring chlorine levels in a stagnant pipe and determining the best fit n and k values for each sampled chlorine decay curve are described in Odimayomi et al. 2026 (see Equations S2-S5).[2]

Equation S2 & S3. Linearized Integrated Rate Law (LIRL) Chloramine Decay Model

$$\frac{C^{1-n}}{1-n} = -kt + \frac{C_0^{1-n}}{1-n} \quad \text{for } n \neq 1 \qquad (2)$$

$$nC = -kt + lnC_0 \quad \text{for } n = 1 \qquad (3)$$

Equation S4 & S5. Nonlinear Least Squares (NLS) Chloramine Decay Model

$$C = \left((n-1)kt + C_0^{1-n}\right)^{\frac{1}{1-n}} \quad \text{for } n \neq 1 \qquad (4)$$

$$C = C_0 e^{-kt} \quad \text{for } n = 1 \qquad (5)$$

For Equations S2-S5, C is chlorine concentration at time t (mg/L), n is the reaction order, C_0 is the initial chloramine concentration (mg/L), and k is the chlorine reaction rate coefficient (variable units dependent on n). LIRL modeling was performed in Excel with k fixed as the inverse of the slope. NLS modeling was done using the Gauss-Newton algorithm in RStudio version 4.4.1.

SI 4. Disinfectant Decay in Glass Jars

Blacksburg tap water was breakpoint chlorinated to a free chlorine residual of 3 mg/L. This water was split into three aliquots: 1) breakpoint chlorinated water inoculated with 4% v/v of GAC influent (182 total cells/mL), 2) breakpoint chlorinated water with the addition of ammonia to a chloramine residual of 6.84 mg/L as Cl_2 (4:1 Cl_2:N weight ratio) and 4% v/v of GAC influent, and 3) chloraminated water without the GAC inoculum. Forty-five 250-mL borosilicate glass jars were prepared by soaking in a 10% nitric acid bath for 12 hours before being rinsed with RO water and baked for 3 hours at 550 °C. Water from each condition was poured into fifteen jars and stored in triplicate at 5, 19, 24, 30, and 39 °C. Jars were kept in the dark rotating at 50 rpm on orbital shakers. Free chlorine and monochloramine were measured in respective jars every 2-3 days over the 20-day experiment.

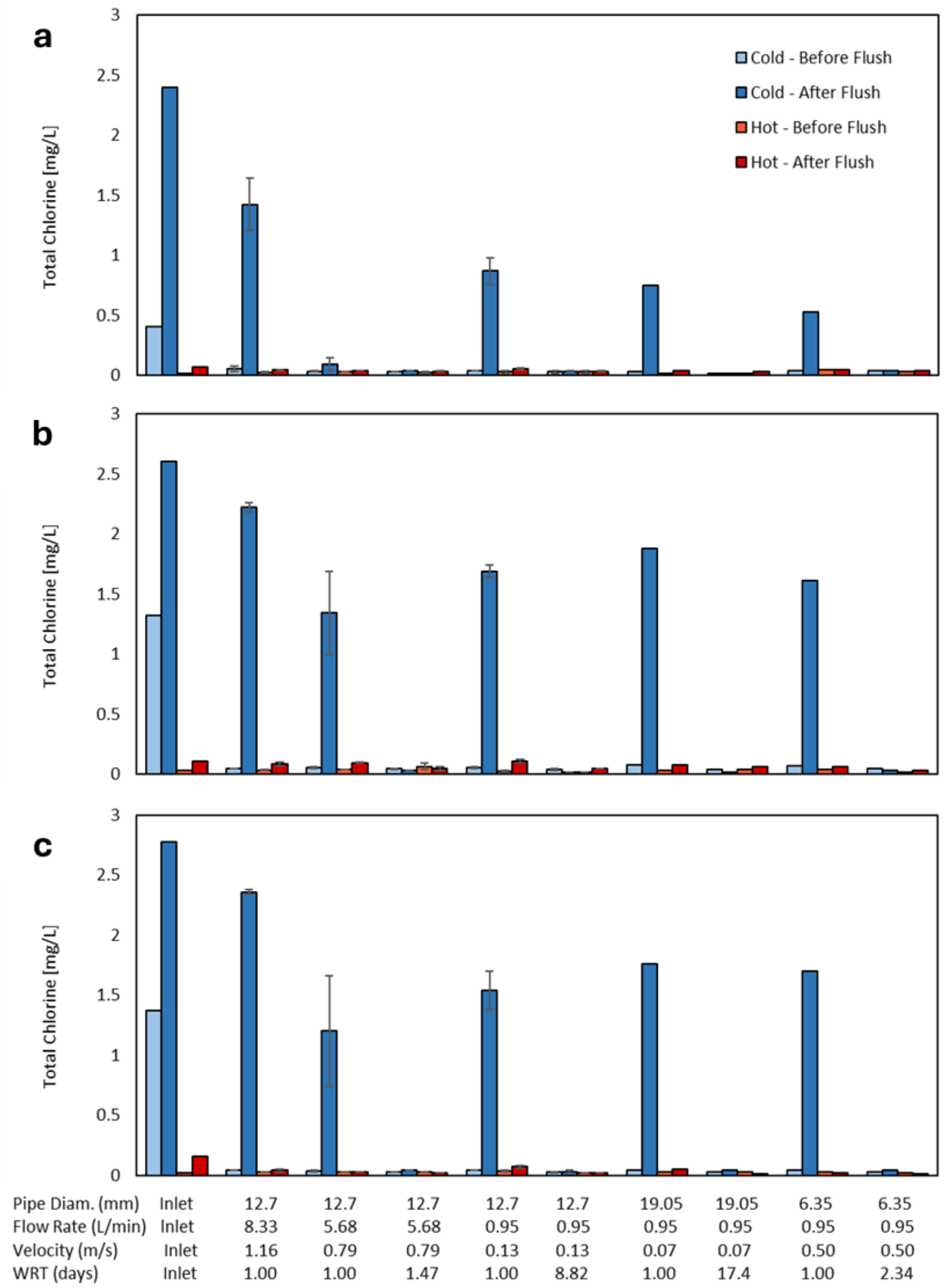

Figure S3. Chloramine Residual Before and After Flush. Chloramine concentration at all cold and hot water taps before and after the daily flush cycle following a) 3 weeks, b) 13 weeks, and c) 23 weeks of treatment at the maximum influent chloramine residual of ~2.5 mg/L. Error bars show the standard deviation of duplicate taps.

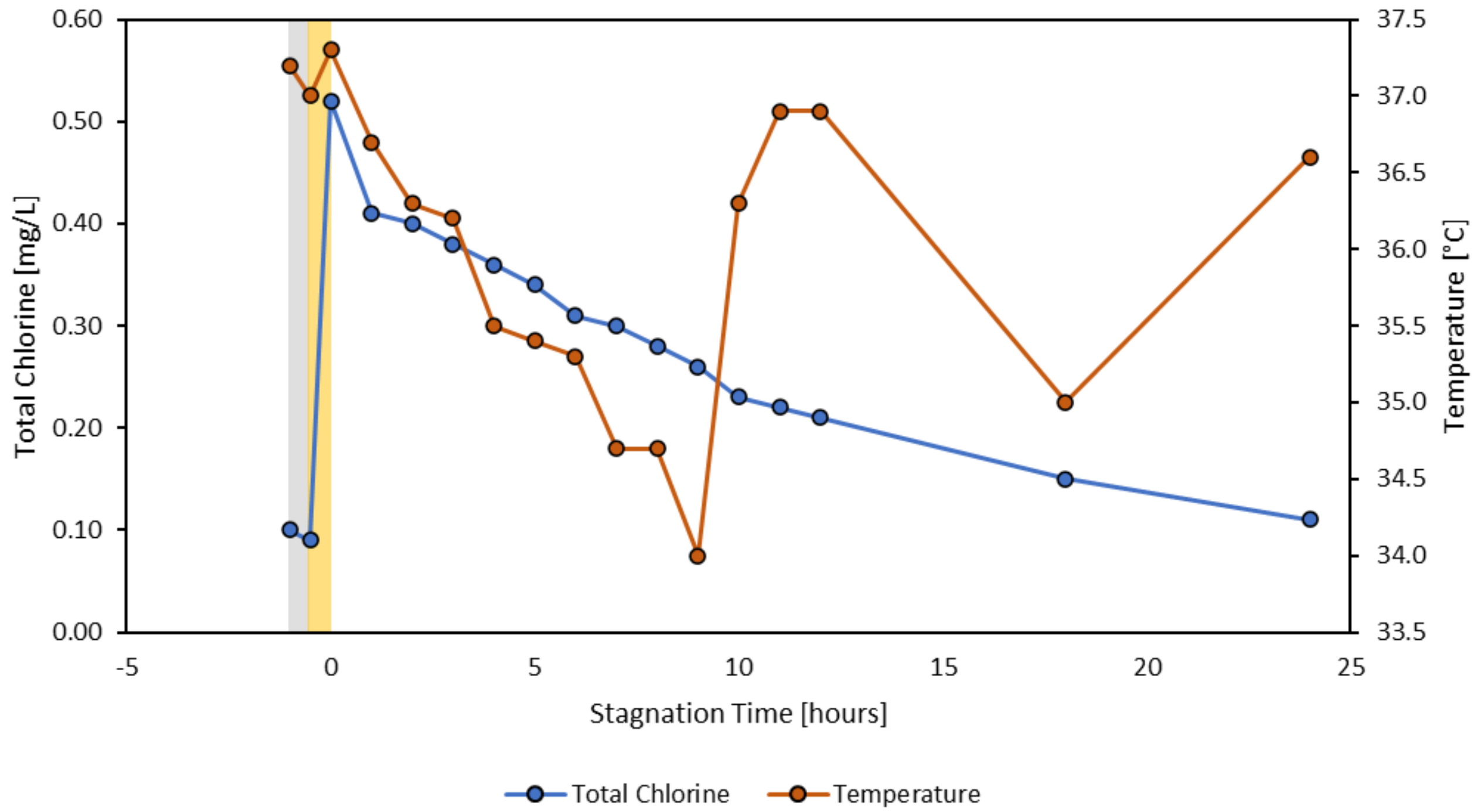

Figure S4. Water Heater Disinfectant Profile. Chloramine (measured as total chlorine) and temperature profiles in the water heater over 24 hours. Gray shaded area: daily hot water pipes flushing event. Yellow shaded area: tank heating element turned on for 30 minutes immediately following the flushing event.

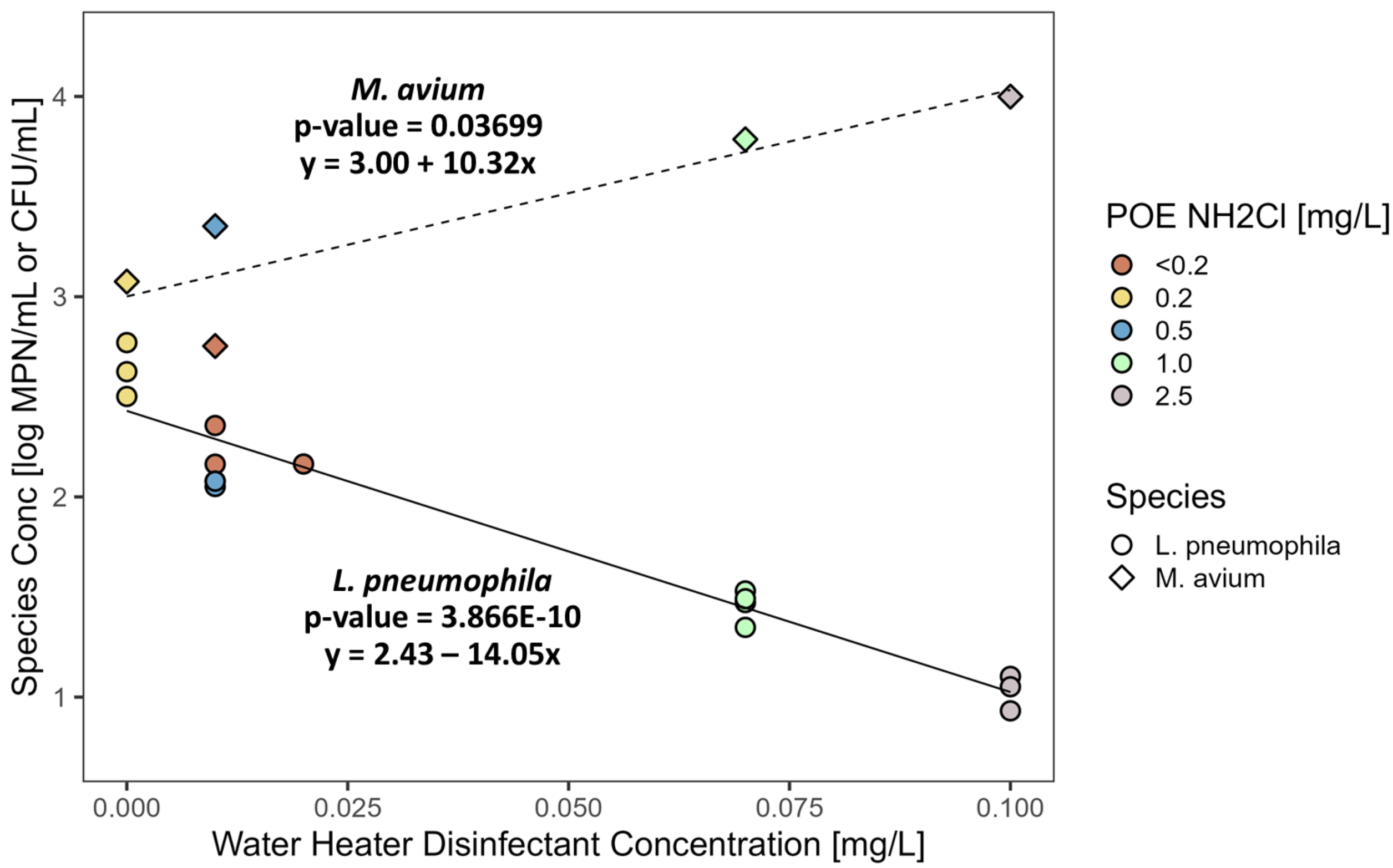

Figure S5. Water Heater Pathogen Trends as a Function of Chloramine Concentration. Culturable *L. pneumophila* (in log MPN/mL) and *M. avium* (in log CFU/mL) trends and total chlorine concentration in the rig 19-gallon electric water heater as influent chloramine concentration increased. Solid line: linear model of *L. pneumophila* in tank. Dotted line: linear model of *M. avium* in tank. POE: point-of-entry.

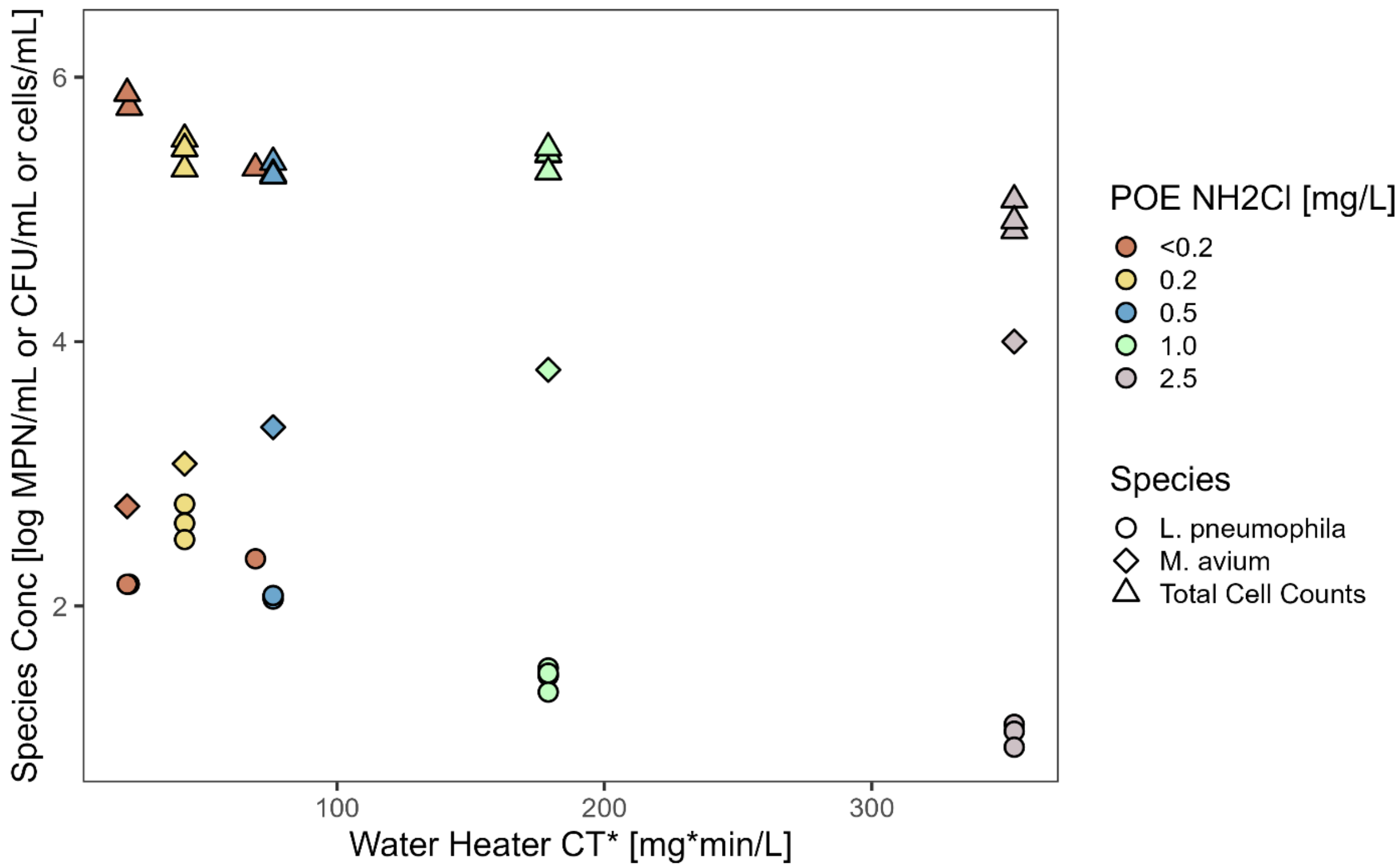

Figure S6. Water Heater Pathogen and Total Cell Trends with PPCT. *L. pneumophila* (in log MPN/mL), *M. avium* (in log CFU/mL), and total cell counts (TCC) (in log cells/mL) trends with CT* values in the rig 72 L electric water heater as influent chloramine concentration increased. CT* values were estimated based on residual measured in the tank 24-hours after flushing and at the rig influent. Chloramine at each influent concentration was fit to a first order decay (k=0.0622 day^{-1}) or n=1.7 order decay (k=0.0715 $L^{0.7}$ $mg^{-0.7}day^{-1}$) based on decay kinetics calculated in the water heater at a 2.5 mg/L influent chloramine concentration (Figure). POE: point-of-entry.

Table S2. Modeled Best Fit n^{th} Order Chloramine PPCT Percent Error from Measured PPCT

Pipe Diameter (mm), Flow Rate (L/min), Replicate[a]	CT Error in Cold 1-Day WRT Taps			
	Target Influent Cl_2 = 0.5 mg/L (1.5 Months)	Target Influent Cl_2 = 1.0 mg/L (2 Months)	Target Influent Cl_2 = 2.5 mg/L (3.5 Months)	Target Influent Cl_2 = 2.5 mg/L (6.5 Months)[b]
**Linear Integrated Rate Law**				
12.7, 8.3, R1	-5%	-31%	-20%	2%
12.7, 8.3, R2	-31%	-33%	-8%	9%
12.7, 5.7, R1	3%	-41%	-70%	-74%
12.7, 5.7, R2	-34%	-35%	-71%	-62%
12.7, 0.95, R1	-33%	-24%	-13%	-40%
12.7, 0.95, R2	-34%	-30%	-75%	-62%
19.05, 0.95	NM[c]	-21%	10%	17%
6.35, 0.95	NM	-16%	-82%	-64%
Average ± Std Dev	-22% ± 15%	-29% ± 7%	-41% ± 34%	-34% ± 35%
**Nonlinear Least Squares**				
12.7, 8.3, R1	-10%	-10%	-2%	12%
12.7, 8.3, R2	-17%	-9%	-2%	14%
12.7, 5.7, R1	-8%	-11%	-13%	0%
12.7, 5.7, R2	-12%	-10%	-11%	4%
12.7, 0.95, R1	-15%	-7%	-7%	5%
12.7, 0.95, R2	-13%	-7%	-7%	5%
19.05, 0.95	NM	-3%	-4%	10%
6.35, 0.95	NM	-13%	--	--
Average ± Std Dev	-12% ± 3%	-9% ± 3%	-7% ± 4%	7% ± 5%

[a]The ability to replicate pipe conditions was limited by plumbing manifold ports.
[b]Due to fluctuations in the disinfectant residual delivered to the building, the influent chloramine concentration was 2.93 mg/L on this day of sample collection. The starting residual was adjusted to 2.5 mg/L to remove the impact of this abnormally high influent chloramine concentration.
[c]NM = not measured
Negative values (orange) are underpredictions and positive values (blue) are overpredictions of CT

References:

(1) Rhoads, W. J.; Keane, T.; Spencer, M. S.; Pruden, A.; Edwards, M. A. Did Municipal Water Distribution System Deficiencies Contribute to a Legionnaires' Disease Outbreak in Quincy, IL? *Environ. Sci. Technol. Lett.* **2020**, *7* (12), 896–902. https://doi.org/10.1021/acs.estlett.0c00637.

(2) Odimayomi, T. O.; Snead, D. C.; Pruden, A.; Edwards, M. A. Complexity of Chloramine Decay Kinetics in Premise Plumbing. *ACS ES&T Water* **2026**. https://doi.org/10.1021/acsestwater.5c01339.
